# A Near Infrared Spectroscopy (NIRS) and Chemometric Approach to Improve Apple Fruit Quality Management: A Case Study on the Cultivars “Cripps Pink” and “Braeburn”

**DOI:** 10.3390/molecules200813603

**Published:** 2015-08-24

**Authors:** Daniela Eisenstecken, Alessia Panarese, Peter Robatscher, Christian W. Huck, Angelo Zanella, Michael Oberhuber

**Affiliations:** 1 Laimburg Research Centre for Agriculture and Forestry, Laimburg 6—Pfatten (Vadena), Auer (Ora) 39040, BZ, Italy; E-Mails: daniela.eisenstecken@provinz.bz.it (D.E.); alessia.panarese@provinz.bz.it (A.P.); peter.robatscher@provinz.bz.it (P.R.); angelo.zanella@provinz.bz.it (A.Z.); 2 Institute of Analytical Chemistry and Radiochemistry, CCB—Center for Chemistry and Biomedicine, Leopold-Franzens University, Innrain 80–82, Innsbruck 6020, Austria; E-Mail: christian.w.huck@uibk.ac.at

**Keywords:** *Malus x domestica* “Braeburn”, *Malus x domestica* “Cripps Pink”, near infrared spectroscopy, NIRS, internal quality, firmness, sugars, apples

## Abstract

The potential of near infrared spectroscopy (NIRS) in the wavelength range of 1000–2500 nm for predicting quality parameters such as total soluble solids (TSS), acidity (TA), firmness, and individual sugars (glucose, fructose, sucrose, and xylose) for two cultivars of apples (“Braeburn” and “Cripps Pink”) was studied during the pre- and post-storage periods. Simultaneously, a qualitative investigation on the capability of NIRS to discriminate varieties, harvest dates, storage periods and fruit inhomogeneity was carried out. In order to generate a sample set with high variability within the most relevant apple quality traits, three different harvest time points in combination with five different storage periods were chosen, and the evolution of important quality parameters was followed both with NIRS and wet chemical methods. By applying a principal component analysis (PCA) a differentiation between the two cultivars, freshly harvested *vs.* long-term stored apples and, notably, between the sun-exposed *vs.* shaded side of apples could be found. For the determination of quality parameters effective prediction models for titratable acid (TA) and individual sugars such as fructose, glucose and sucrose by using partial least square (PLS) regression have been developed. Our results complement earlier reports, highlighting the versatility of NIRS as a fast, non-invasive method for quantitative and qualitative studies on apples.

## 1. Introduction

Apple fruit quality is evaluated by external appearance using optical sensors on sorting machines and by destructive methods to measure internal quality traits. Fruit maturation and storage are known to influence the chemical composition, and therefore, the quality of apple fruit [1,2,3,4]. To meet consumers’ expectations for excellent fruit quality, detailed knowledge of several quality parameters during the harvest period and post-harvest management [5] are required. Minimum values for the most established quality parameters, including fruit flesh firmness, sugar content, and acidity, are increasingly demanded by national and EU legislation [6,7]. The choice of the harvest timepoint has a major effect on the preservation of quality during storage [3,8]. Non-destructive spectroscopic methods, electronic noses and electronic tongues are experiencing growing interest [9,10,11] in food science in recent years, because they are fast, easy to use, reagent free, and compatible with in-line and on-line measurement systems. Among these analytical techniques near infrared spectroscopy (NIRS) has attracted much attention [12,13,14,15,16,17]. Chemometric approaches were successfully used to develop prediction models for various quality parameters, including sugar and acid content, firmness, physiological disorders such as brownheart, but also authentication issues were addressed effectively [9,18,19]. However, NIR-based prediction models are not always available for the desired parameters and specific apple cultivars. In this study, we have explored the potential of NIRS to identify harvest time points, storage times, fruit inhomogeneity, and to build up prediction models for individual sugars as well as other quality parameters for “Cripps Pink” and “Braeburn” apples. Both cultivars have a firm fruit flesh with different fruit flesh softening behavior during storage. Moreover, no comprehensive data have been reported on the chemical composition of the two cultivars as well as on the spectral evaluation of the quality parameters including different harvest time-points and storage periods.

## 2. Results and Discussion

### 2.1. Diversity of the Sample Set: Relevant Quality Parameters of “Braeburn” and “Cripps Pink” Apples Considering Three Different Harvest Time-Points and Their Evolution during Long-Term CA Storage

In order to obtain a representative sample set for each cultivar with the widest possible spread of parameters three harvest time points and five different storage periods were selected. The analysis of the sample set was carried out on established quality parameters including starch index (SI), titratable acidity (TA), total soluble solids (TSS), five penetrometric parameters (F_f_, D, W_f_, S, and F_LC_), and individual sugars (fructose, glucose, sucrose, and xylose) which were later used to develop NIR prediction models. Nine parameters were determined at harvest for both cultivars investigated in this study. Significant differences (*p* < 0.05) between the harvest dates were found for SI, TA, TSS, deformation associated with total puncture force (D), work associated with total puncture force (W_f_), flesh limit compression force (F_LC_), and slope of the force-deformation curve (S) in “Braeburn” apples and for SI, TA, total puncture force (F_f_), W_f_, and F_LC_ in “Cripps Pink” apples (Table 1), respectively.

**Table 1:** Quality parameters (mean ± standard deviation) determined at harvest in “Braeburn” and “Cripps Pink” apples.

Cultivar	“Braeburn”			“Cripps Pink”		
Harvest Time Point	HT1	HT2	HT3	HT1	HT2	HT3
**sample number**	30	30	30	30	30	30
**starch index** *	2.7 ± 0.4 ^b^	3.5 ± 0.7 ^a^	3.7 ± 0.6 ^a^	2.8 ± 0.3 ^a^	3.0 ± 0.2 ^a^	3.5 ± 0.2 ^b^
**weight [g]** *	206.6 ± 40.1	209.5 ± 31.7	208.8 ± 33.2	208.3 ± 33.4	211.1 ± 33.7	215.4 ± 23.4
**pH ^#^**	3.55 ± 0.06	3.54 ± 0.08	3.58 ± 0.08	3.51 ± 0.05	3.49 ± 0.06	3.49 ± 0.04
**TA [g/L malic acid] ^#^**	5.6 ± 0.5 ^a^	5.3 ± 0.9 ^a^	4.6 ± 0.7 ^b^	5.6 ± 0.6 ^a^	5.1 ± 0.5 ^b^	5.4 ± 0.4 ^a^
**TSS [°Brix]** *	10.6 ± 3.0 ^a^	9.9 ± 2.6 ^a^	12.2 ± 1.5 ^b^	13.4 ± 0.4	13.3 ± 0.5	13.2 ± 0.5
**F_f_ [N]** *	92.5 ± 9.4	86.7 ± 11.1	87.2 ± 12.3	110.0 ± 10.4 ^a^	105.0 ± 7.5 ^a^	94.4 ± 7.9 ^b^
**D [mm]** *	3.91 ± 0.47 ^a^	3.55 ± 0.26 ^b^	3.68 ± 0.53 ^a,b^	5.15 ± 0.73	4.90 ± 0.68	5.01 ± 0.89
W_f_ [J] *	0.21 ± 0.04 ^b^	0.18 ± 0.03 ^a^	0.18 ± 0.04 ^a^	0.32 ± 0.07 ^a^	0.29 ± 0.06 ^a,b^	0.28 ± 0.06 ^b^
**F_LC_ [N]** *	70.8 ± 6.2 ^b^	63.2 ± 7.1 ^a^	63.8 ± 9.8 ^a^	95.4 ± 6.8 ^a^	92.2 ± 5.1 ^a^	84.2 ± 6.0 ^b^
**S [N/mm]** *	37.6 ± 4.8 ^a,b^	39.6 ± 13.0 ^a^	33.5 ± 5.7 ^b^	37.8 ± 3.6	37.0 ± 4.2	36.9 ± 4.9

Results with different superscript letters in the same row differ significantly (*p* < 0.05) within one cultivar; * ANOVA followed by the Tukey test; ^#^ Kruskal-Wallis test followed by the Mann-Whitney *U* test with Bonferroni correction.

In general, a different behavior during the harvesting period was observed in both cultivars. “Braeburn” apples showed a significant difference (*p* < 0.05) between HT1 and the later harvest time-points for SI, W_f_, and F_LC_, whereas “Cripps Pink” apples showed a significant difference (*p* < 0.05) between the earlier harvest dates and HT3 for SI, F_f_, and F_LC_. It is well established that ripening is associated with starch degradation [20], increased ethylene production [21] thus leading to a softening of the fruit flesh firmness [22], and a decrease in titratable acidity [23]. The monitored losses in titratable acidity and fruit firmness found in the present study are in line with studies reported by Shafiq *et al.* [24] for “Cripps Pink”, Johnston *et al.* [22] for “Royal Gala” and Zhang *et al.* [2] for “Honeycrisp” apples. TSS remained relatively unaffected during the three picking dates, which is consistent with results found in other studies [24,25].

The freshly harvested apples were stored for 0–32 weeks for “Braeburn” apples and 0–30 weeks for “Cripps Pink” apples, and the evolution of fourteen quality parameters was followed during storage (Table 2, Supplementary Tables S1 and S2). Significant differences (*p* < 0.05) for HT1 for almost all parameters, including total extracted juice, pH, TA, TSS, all penetrometric parameters, xylose, and sucrose in both “Braeburn” and “Cripps Pink” apples, and additionally glucose in “Cripps Pink” apples, were observed. Similar results were found during the analogous storage of apples harvested at suboptimal time points (HT2 and HT3). The postharvest evolution of the observed parameters is in line with previous studies, showing a decrease in acidity due to metabolism [12,26] and in firmness significantly depending on rate of evapotranspiration and respiration [1,26] and the disassembly of primary cell wall and middle lamella structures [27], respectively.

**Table 2:** Post-harvest evolution of quality parameters (mean) of “Braeburn” and “Cripps Pink” apples from the optimum harvest date (HT1) during long-term CA storage.

Cultivar	“Braeburn”						“Cripps Pink”					
**CA storage [weeks]**	**0**	**7**	**15**	**21**	**28**	**32**	**0**	**6**	**15**	**20**	**27**	**30**
**sample number**	30	30	26	26	26	28	30	30	30	30	30	28
**weight [g]** *	206.6	208.7	196.9	197.3	199.7	204.0	208.3	212.3	197.9	202.3	199.6	201.3
**total juice [mL]** *		125.7 ^a^	122.7 ^a^	102.2 ^b^	110.0 ^a,b^	120.5 ^a,b^		130.0 ^a^	116.9 ^a,b^	103.5 ^b,c^	98.9 ^c^	111.3 ^b,c^
**pH ^#^**	3.55 ^a^	3.49 ^c^	3.57 ^a^	3.66 ^b^	3.68 ^b^		3.51 ^a^	3.60 ^b^	3.74 ^c^	3.70 ^d^	3.79 ^e^	3.84 ^f^
**TA [g/L malic acid] ^#^**	5.6 ^a^	5.6 ^a^	5.1 ^b^	4.6 ^c^	4.5 ^c^		5.6 ^a^	4.8 ^b^	4.0 ^c^	4.0 ^c^	3.6 ^d^	3.6 ^d^
**TSS [°Brix]** *	10.6 ^b^	13.0 ^a^	12.9 ^a^		12.9 ^a^		13.4 ^a^	12.6 ^a,b,c^	12.9 ^b,c^	12.4 ^c^	13.0 ^a,b,c^	13.2 ^a,b^
**F_f_ [N]** *	92.5 ^a^	93.4 ^a^	84.0 ^b^	78.5 ^b,c^	76.2 ^c^	75.9 ^c^	110.0 ^a^	91.6 ^b^	85.9 ^b,c^	87.1 ^b,c^	81.2 ^c^	
**D [mm]** *	3.91 ^a^	3.62 ^a,b,c^	3.48 ^b,c^	3.39 ^b,c^	3.26 ^c^	3.67 ^a,b^	5.15 ^a^	4.65 ^b^	4.52 ^b,c^	4.45 ^b,c^	4.22 ^c^	
**W_f_ [J]** *	0.21 ^a^	0.19 ^a,b^	0.17 ^b,c^	0.16 ^c^	0.14 ^c^	0.16 ^b,c^	0.32 ^a^	0.24 ^b^	0.22 ^b,c^	0.22 ^b,c^	0.19 ^c^	
**F_LC_ [N]** *	70.8 ^a^	67.5 ^a,b^	65.1 ^b,c^	60.0 ^c,d^	58.1 ^d^	57.7 ^d^	95.4 ^a^	74.2 ^b^	63.5 ^c^	63.0 ^c,d^	58.3 ^d^	
**S [N/mm]** *	37.6 ^a,b^	38.4 ^a,b^	35.9 ^a^	38.5 ^a,b^	42.4 ^b^	36.3 ^a,b^	37.8 ^a,b^	32.4 ^a^	49.5 ^b^	31.1 ^a^	30.4 ^a^	
**glucose [g/100 g] ^#^**		1.1	1.2	1.1	1.0	1.2		0.5 ^a^	0.5 ^a^	0.7 ^a,b^	0.9 ^b^	0.6 ^a,b^
**xylose [g/100 g]** *		0.03 ^b^	0.05 ^a^	0.06 ^a^	0.05 ^a^	0.06 ^a^		0.03 ^a^	0.03 ^a^	0.05 ^a,b^	0.06 ^b^	0.06 ^b^
**sucrose [g/100 g] ^#^**		2.9 ^a^	3.2 ^a^	1.7 ^b^	1.2 ^b,c^	0.9 ^c^		3.5 ^a^	3.0 ^a,b^	2.4 ^b^	2.6 ^b^	2.4 ^b^
**fructose [g/100 g]** *		2.6	2.3	2.2	2.2	2.1		3.1	2.7	2.5	3.2	2.8

Means with different superscript letters in the same row differ significantly (*p* < 0.05) within one cultivar; * ANOVA followed by the Tukey test; ^#^ Kruskal-Wallis test followed by the Mann-Whitney *U* test with Bonferroni correction.

Surprisingly, our study exhibited non-significant variations in TSS, even though an increase is rather expected due to the starch breakdown [28] or the hydrolysis of cell wall polysaccharides [29]. Fructose and sucrose were identified as the principal saccharides in both cultivars, whereas glucose was found generally higher in “Braeburn” than in “Cripps Pink” apples. Sucrose showed a significant decrease during storage, whereas xylose, present only in trace amounts, increased significantly with storage. Interestingly, fructose did not show a clear post-harvest trend in both cultivars, but fluctuated throughout the whole storage period. In summary our experimental design of the study yielded in a diverse sample set with high variability within the most relevant apple quality traits, which was submitted to NIR spectroscopic analysis.

### 2.2. Qualitative Analysis by Means of Near Infrared Spectroscopy

NIR spectra from 1049 apples (“Braeburn” and “Cripps Pink”) were acquired and submitted to principal component analysis (PCA) for an evaluation of differences related to variety, harvest time points and storage time points. Four spectra per fruit were averaged and subjected to de-trending followed by first derivative Savitzky-Golay nine points [30] (derivative order: 1, polynomial order: 2) (Supplementary Information, Figure S1).

**Figure 1:**
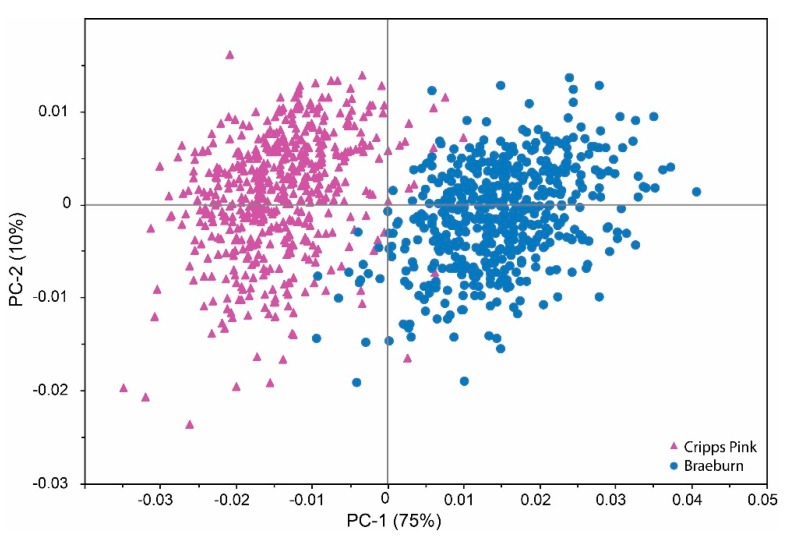
PCA score plot NIR data acquired from the complete data set (515 “Braeburn” and 534 “Cripps Pink” apples).

In order to investigate the effect of the cultivar on spectral data, PCA was performed on the whole dataset consisting of 515 “Braeburn” and 534 “Cripps Pink” apples. Principal components (PC) 1 and 2 accounted for 75% and 10% of the total variance, respectively (Figure 1).

Both cultivars are nearly separated on the first PC even though the dataset consists of different harvest dates and storage times. Loading plots (Supplementary Information, Figure S2) for NIR data are difficult to interpret regarding the influence of individual metabolites of a food sample on the separation. However, the wavelength regions from 1400 nm to 1420 nm, 1850 nm to 1940 nm, and 1960 nm to 2045 nm contribute most to the distinction power of the model. Signals in the first region arise to the first overtone of O-H bonds of sugars and water, whereas the second and third belongs to O-H combination bands [31]. Indeed, significant differences (ANOVA: *p* < 0.05) were found in firmness, TA, TSS, fructose, glucose and sucrose (data not shown) between the two varieties. This suggests that both cultivars might principally be separated from each other on the basis of differences in sugar and polysaccharide composition [15].

The next step was to test whether NIRS was able to differentiate harvest time points. The PCA of the NIR spectra showed no differentiation between the three harvest dates for both cultivars. Also, applying a PCA to the wet-chemical data without starch index from the harvest samples was unable to separate the harvest dates, confirming subtle metabolic differences between HT1 to HT3. Similar results regarding the uniformity in the major quality parameters (firmness, TSS and TA) were found by McGlone *et al.* [17] during a period of three weeks before and one week after the commercial harvest date. However, they found a significant reduction of the chlorophyll absorbance peak at 680 nm during their harvesting period. Also, Zanella *et al.* [32] showed that it is possible to discriminate among different harvest dates using non-destructive optical indices based on the chlorophyll content of apple peels. Therefore, we conclude that the metabolic differences in apples from different harvest dates cannot be detected in NIR spectra but require other methods or regions of the electromagnetic spectrum.

**Figure 2:**
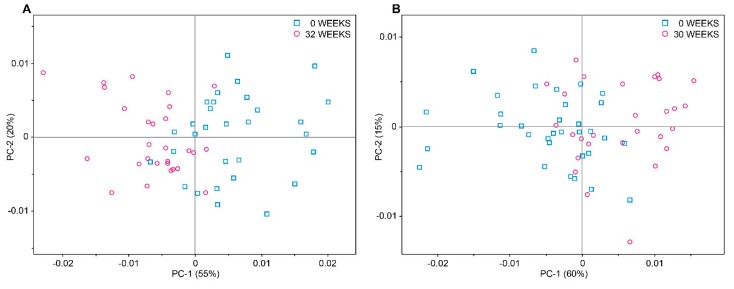
PCA score plot of NIR data acquired from freshly harvested (0 weeks) and long-term stored (32/30 weeks) apples from the optimal harvest date (HT1). (**A**) “Braeburn” (**B**) “Cripps Pink”.

Next a PCA was performed on a subset of NIR spectra, comprising freshly harvested (0 week storage) and long-term stored (32 and 30 weeks storage for “Braeburn” and “Cripps Pink”, respectively) apples from the optimal harvest date (HT1). Figure 2 shows a differentiation tendency for both cultivars on the first PC; for “Braeburn” apples (Figure 2A) the trend was more pronounced than for “Cripps Pink” apples (Figure 2B).

The differentiation was linked to spectral regions arising from the O-H combination band (around 2000 nm), the 2nd overtone of the C=O stretch (around 1890 nm) and the 1st overtone of C-H combination bands (around 1400 nm), which can be attributed to the differences in sugar and acid content between freshly harvested and long-term stored apples [12,23,26,29]. There is no direct evidence to support the better differentiation of “Braeburn” apples in the PCA, but it is well established that metabolic changes during storage (ripening) are cultivar dependent. For instance, Ng *et al.* [33] showed a different response of various cultivars in reducing density of cell packing and increasing cell volume and air spaces, and Gwanpua *et al.* [34] found different losses of site chains neutral sugars from pectin during storage, resulting in variety-specific cellular and intercellular structures, thus influencing the light propagation through the apple tissue and affecting the scattering events [35,36]. When the subset was expanded to all time points during storage the pattern along PC 1 was evident, but no clear separation between the time points was achieved.

**Figure 3:**
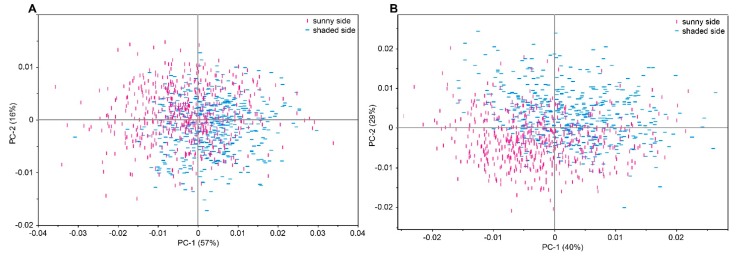
PCA plot of all apple NIR spectral data acquired from the sunny and shaded side of the apples (**A**) 511 “Braeburn” apples on both sunny and shaded side (**B**) 539 “Cripps Pink” for the sunny side and 533 apples for the shaded side.

In addition we evaluated whether NIR spectra could identify the sun-exposed side of apple fruits, by measuring four points on the sun-exposed and four points on the shaded side. Li *et al.* [37] compared the primary and secondary metabolism in the sun-exposed peel and the shaded peel of apple fruit. They found significant differences in the respiratory metabolism and in the phenylpropanoid pathway between the two apple sides mainly due to different peel temperature and solar irradiance. PCA was performed on the whole dataset consisting of samples from all three harvest dates and all six storage time points for each cultivar. The first two PCs accounted for 74% of the total variance in “Braeburn” and 69% in “Cripps Pink” apples, respectively. Figure 3 shows a tendency to separate the sun-exposed from the shaded side of apples in both cultivars despite the broad diversity due to different harvest dates and storage times. In both cultivars the wavelength region from 1870 to 1920 nm contributed significantly to the observed trend, which can be linked to the O-H combination band of water and the first overtone of C-H combination bands. A further major influence on the PCA model was exerted by the wavelengths from 2000 to 2300 nm, which can be interpreted as combination bands of N-H and O-H bonds from sugars, polysaccharides and amino acids found in higher levels in sun-exposed apple peel [37]. Even though these spectral regions provide some information on the possible molecular background of the observed differences, more research would be required to understand the relevant metabolite classes.

### 2.3. Development of Multivariate Calibration Models

NIRS has successfully been used for quantitative analysis in complex matrices such as wine [38], natural products [16] and fruit and vegetables [9]. In our dataset, we first selected the most promising variables among the wet chemical parameters based upon variation and range within the dataset (Table 1, Table 2, Table S1, and Table S2). Various data processing techniques were tested to optimize the prediction model of each selected parameter, applied on the single cultivar or the combination of both. Table 3 shows only the data treatment yielding in the best prediction models for each parameter, considering the penetration depth of NIR radiation into fruit tissue is only a few millimeters [39], frequently limiting the prediction of fruit quality attributes in apples by NIRS. The accuracy of the prediction models was validated by the random division of the data set into a training set (2/3 of the data) and a test set (1/3 of the data) for the large data sets of the major quality parameters (TSS, TA, pH, F_f_, D, W_f_, F_LC_). The assignment of samples to the two subsets was carried out using an implemented algorithm in the NIRCal^©^ software that divides samples into blocks. For individual sugars the cross validation method using the leave-one-out algorithm was chosen due to the limited sample numbers [40].

Generally, the best calibration models for TSS and TA were achieved after a simple two-step data pre-treatment using a normalization to reduce baseline variations and prevent light scattering effects and the first derivative to allow correction of linear offsets and to increase smaller absorption peaks. The first derivative BCAP is performed on each absorption value at wavenumber *i* using the following equation:

(1) $$f^{ʹ}(x_{i})=(f(x_{i+2})+f(x_{i+1})-f(x_{i-1})-f(x_{i-2}))/4$$

For the prediction of pH, using a normalization was sufficient. For all parameters a selection of wavelength ranges was carried out. As shown in Table 3 the coefficients of determination found for TA and pH resulted in similar or better correlation coefficients, SECs and SEPs compared to literature [13,17,35]. This improvement may also be attributed to a wider range of the values found in this study. Notably, calibration models developed for “Cripps Pink” apples showed better performance than those for “Braeburn” apples in terms of coefficients of determination of calibration and validation. The cultivar “Braeburn” had generally higher absorbance, and thus lower reflectance compared to “Cripps Pink”. Both cultivars showed higher absorbance in riper apples, a result that has been already shown [35,36]. In general, when comparing the TSS and TA contents along with the firmness of both cultivars, “Braeburn” apples were rather soft, lower in TSS content and higher in acidity. Thus the chemical and textural differences could explain the different behavior of the cultivars in the PLS regression analysis and the PCA (Figure 1).

The TSS prediction model showed lower to inadequate coefficients of determination in contrast to previous studies [13,17,35]. This might be attributed to the small range of TSS values of only about 4 °Brix compared to those found in the above mentioned literature of about 8 to 10 °Brix. Additionally, Peirs *et al.* [35] and McGlone *et al.* [17] included wavelength areas in the VIS range to increase the model accuracy. Contrary to the results found for TA and pH, the r values for “Cripps Pink” apples were very low (r^2^_cal_ = 0.03) compared to “Braeburn” apples (r^2^_cal_ = 0.49). However, the SEC and SEP for the individual apple cultivars are similar to results found in literature [9] ranging between 0.5 and 0.6 °Brix.

**Table 3:** Summary of the best NIR prediction models for the indicated apple quality parameters: “Cripps Pink” (CP), “Braeburn” (BB), the latent variables (LV), the standard error of calibration (SEC), the standard error of prediction (SEP), root mean square error of cross validation (RMSECV), the coefficient of determination (r^2^) referring to validation and calibration, and the bias referring to prediction. The overall range of the wet chemical values, wavelength selections, the data pre-treatments, and the amount of total samples (N) are listed.

Parameters ^#^	Cultivar	Range	Wavelength Selection [nm]	Data Treatment	LV	N	Calibration		Validation		
							SEC	r^2^	SEP	r^2^	Bias
**TSS [°Brix]**	CP	11.3–14.9	1041–2325	n01, 1st derivative BCAP	3	510	0.57	0.03	0.56	0.02	–0.00
	BB	10.0–14.7	1388–2083	1st derivative BCAP, SNV	6	388	0.52	0.49	0.52	0.38	–0.08
	both	10.7–14.6	1111–1351, 1408–2000	1st derivative BCAP, ncl	5	866	0.58	0.15	0.59	0.14	–0.00
**TA [g/L malic acid]**	CP	2.7–6.4	1041–2380	ncl, 1st derivative BCAP	8	533	0.32	0.85	0.44	0.69	–0.04
	BB	3.2–6.5	1136–2272	1st derivative BCAP, MSC full	6	428	0.43	0.52	0.45	0.50	–0.04
	both	2.7–6.8	1000–2000	SNV, 1st derivative BCAP	8	959	0.41	0.74	0.48	0.67	0.06
**pH**	CP	3.39–4.00	1000–2439	ncl	12	533	0.06	0.81	0.06	0.81	0.00
	BB	3.37–3.84	1000–1282, 1515–1851, 2083–2272	ncl	12	428	0.05	0.62	0.05	0.62	0.01
	both	3.37–4.00	1111–2439	SNV	10	959	0.09	0.49	0.09	0.50	–0.00
**F_f_ [N]**	CP	60.8–109.8	1000–2495	none	9	346	9.4	0.11	9.4	0.14	0.05
	BB	49.0–110.8	1086–2325	none	12	494	7.8	0.56	7.9	0.55	–0.03
	both	49.0–124.5	1111–2272	none	9	867	10.8	0.31	9.8	0.29	0.03
**D [mm]**	CP	3.04–7.85	1086–2380	none	14	357	0.69	0.30	0.75	0.29	0.08
	BB	2.59–4.79	1111–2439	none	14	495	0.39	0.18	0.40	0.15	0.00
	both	2.59–7.85	1086–2380	none	14	868	0.66	0.46	0.68	0.45	0.01
**W_f_ [J]**	CP	0.11–0.48	1111–1351, 1408–2000	none	12	358	0.06	0.18	0.06	0.08	0.00
	BB	0.08–0.27	1086–2439	none	11	491	0.03	0.35	0.03	0.38	–0.00
	both	0.08–0.48	1086–2439	none	11	867	0.05	0.32	0.05	0.36	0.00
**F_LC_ [N]**	CP	43.9–101.2	1098–2222	none	13	334	8.7	0.40	9.2	0.24	0.47
	BB	33.9–93.3	1111–2272	none	13	424	6.4	0.50	6.5	0.46	–0.27
	both	33.9–101.2	1063–2272	none	7	758	9.2	0.29	8.6	0.22	–0.30
							**SECV**	**r^2^_CV_**			
**glucose [g/100 g]**	CP	0.3–1.3	1111–2252	SNV. 1st derivative SG 9 points	12	73	0.2	0.85			
	BB	0.6–1.8	1111–2380	SNV. 1st derivative SG 9 points	10	77	0.3	0.79			
	both	0.3–1.8	1063–2272	SNV. 1st derivative SG 9 points	10	150	0.2	0.83			
**xylose [g/100 g]**	CP	0.02–0.08	1111–1351, 1408–2000	1st derivative BCAP. SNV	8	73	0.02	0.81			
	BB	0.01–0.07	1136–2272	1st derivative BCAP. SNV	8	77	0.01	0.76			
	both	0.01–0.08	1063–2272	1st derivative BCAP. SNV	7	150	0.01	0.59			
**sucrose [g/100 g]**	CP	1.4–4.1	1111–2380	ncl. 1st derivative BCAP	10	73	0.7	0.85			
	BB	0.5–3.9	1111–2380	ncl. 1st derivative BCAP	10	77	0.8	0.79			
	both	0.5–4.1	1111–2272	ncl. 1st derivative BCAP	10	150	0.7	0.74			
**fructose [g/100 g]**	CP	1.6–3.8	1111–2272	ncl. 1st derivative SG 9 points	8	73	0.6	0.62			
	BB	0.9–4.3	1111–2272	ncl. 1st derivative SG 9 points	10	77	0.9	0.76			
	both	0.9–4.3	1111–2272	ncl. 1st derivative SG 9 points	10	150	0.7	0.55			

^#^ For the major quality parameters (TSS, TA, pH, F_f_, D, W_f_, and F_LC_ two third of the dataset were used in calibration and one third in validation, while for the carbohydrates cross-validation was used.

PLS analysis of individual sugars by the single varieties showed good coefficients of determination, which were always above 0.55; however, our data do not allow for conclusive statements on the models’ selectivity towards individual sugars, as they are partly intercorrelated in our sample set,. Using a normalization and the first derivative gave more accurate models than the raw data or the second derivative. By performing a PLS regression analysis on the merged dataset, the obtained r^2^ values were generally lower in respect to the single variety models except for glucose. So far, measurements of constituent sugars of intact apple fruit by NIR spectroscopy have been reported only by Liu *et al.* [41] on “Fuji” apples, with excellent concentration ranges for glucose, fructose and sucrose. The range of glucose and fructose in cv. “Fuji” was 1.92–4.50 g/100 g and 4.68–10.41 g/100 g, respectively and hence, much higher than in cv. “Braeburn” and “Cripps Pink”. The range of sucrose is similar to that found in this study. The coefficients of determination and RMSECV reported here are slightly lower, that can be attributed to the smaller range of sugar values. However, our results are not conclusive. In general, these correlations need to be interpreted carefully.

For the penetrometric parameters, the scattering effects and texture properties are very important. As a consequence, the raw spectra were used in the PLS analyses in order to preserve the scattering information. Different wavelengths were selected to build up the calibration models. F_f_ showed an acceptable coefficient of determination for “Braeburn” apples (r^2^ = 0.55) and a poor for “Cripps Pink” apples (r^2^ = 0.11). SEC and SEP were 7.8 and 7.9 N for “Braeburn” and 9.4 and 9.4 N for “Cripps Pink”, respectively, being quite consistent with those reported in literature [14,17].

## 3. Experimental Section

### 3.1. Fruit Material

*Malus x domestica* Borkh. (cultivar “Braeburn” and “Cripps Pink”) were grown at the experimental orchard at Laimburg (Bolzano, Italy, 220 m.a.s.l.) according to the regional guidelines of integrated production [42]. Apples (540 per cultivar) were picked at three different harvest time points (HT1 = optimal harvest date, HT2 = one week after HT1, and HT3 = two weeks after HT1), and each harvest time point was randomly divided into six batches of 30 fruit and stored under ultra-low oxygen (ULO) conditions (1.5 kPa O_2_ and 1.3 kPa CO_2_) for 7, 15, 21, 28 and 32 weeks for “Braeburn” at 1.3 °C and for 6, 15, 20, 27 and 30 weeks for “Cripps Pink” at 2.5 °C, respectively. After storage each single fruit was inspected visually for storage damages and 31 apples showing bruises and mold were excluded from the study. The remaining 1049 apples were measured at the different harvest and storage times first non-destructively by near infrared spectroscopy (NIRS) and then destructively to assess firmness. The apples were then pressed individually to gain the juice for determining the other wet chemical parameters. The apple juice was immediately frozen at −80 °C after production and stored until further analysis.

### 3.2. NIRS

NIR spectra were recorded in diffuse reflectance mode with a Buchi NIRFlex^©^ N-500 FT-NIR spectrometer, the Fibre Optic Solids cell and NIRWare^©^ 1.4.3010 software package (Buchi^®^ AG, Flawil, Switzerland). Wavelengths from 10,000 to 4000 cm^−1^ (1000–2500 nm) were acquired with a resolution of 4 cm^−1^, an absolute wavenumber accuracy of ±2 cm^−1^ and a relative reproducibility of 2.0 cm^−1^. The number of scans was 4 × 32 for each point measurement. Four point measurements equally distributed around the equator, four on the sun-exposed and four on the shaded side were performed. Internal and external reference measurements were repeated every hour (external reference against a Spectralon^®^ assembled reference cap). A bifurcated fiber optic probe of 2 m length with enclosed fiber bundles of 2.0 mm diameter (light beam) and 3.5 mm diameter (light collector) was used.

### 3.3. Physicochemical Parameters

#### 3.3.1. Standards

All standards and chemicals of analytical grade were purchased from Sigma-Aldrich (St. Louis, MO, USA).

#### 3.3.2. Starch Index

The starch degradation was determined by cutting from each apple a 1 cm thick disk at equatorial level and then dipped for one minute in Lugol’s reagent (10 g·L^−1^ KI + 3 g·L^−1^ I_2_ in H_2_O). The maturity stage expressed as Starch index (SI) was then visually assessed by comparison with the 1 (100% starch) to 5 (0% starch) color chart proposed by Laimburg Research Centre for apples [43].

#### 3.3.3. Firmness

Fruit firmness was measured by the TA Plus Texture Analyzer (Lloyd Instrument, West Sussex, UK) leading to a force-deformation curve which gives a more accurate description of texture. An 11 mm plunger was used for penetration into the apple flesh until a depth of 8 mm with a speed of 200 mm/min. The apple skin was not removed [44] and the curve was recorded both on the sunny and the shaded side of the apple. From the force-deformation curve four parameters were calculated: total puncture force (F_f_,), deformation associated with total puncture force (D), work associated with F_f_ (W_f_), slope of the force-deformation curve (S) and flesh limit compression force (F_LC_).

#### 3.3.4. Total Soluble Solids

The total sugar content was measured as total soluble solids (TSS, expressed as °Brix) on the extracted and filtered (Ø 185 mm, Macherey-Nagel, Düren, Germany) apple juice with a refractometer (Bellingham and Stanley, Kent, UK) at 20 °C.

#### 3.3.5. Titratable Acid and pH

The total acidity was measured in freshly prepared juice. Titratable acidity (TA) was determined using automatic titrator Titromatic 1S (Crison, Barcelona, Spain) by titration of 35 mL of juice with 0.33 M NaOH to the end point at pH = 8.2. The results were expressed as g/L malic acid. At the same time the titration device determined the pH of samples with a previously calibrated pH electrode.

#### 3.3.6. Extraction and Individual Sugar Determination

Individual sugars (fructose, glucose, sucrose, and xylose) were analyzed using an ion chromatograph with pulsed amperometric detection (HPAE-PAD). The instrument was a ICS-5000 (Thermo Scientific Dionex, Sunnyvale, CA, USA) using a Dionex CarboPac PA1 Analytical column (4 × 250 mm) and a Dionex CarboPac PA1 Guard column (4 × 50 mm). Separation of sugars was achieved by isocratic elution with 10 mM sodium hydroxide (NaOH) and the column was regenerated with 200 mM NaOH for 10 min. Flow rate was set at 1.0 mL/min, injection volume at 20 μL and column temperature at 30 °C; an Au on PTFE disposable working electrode and a pH-Ag/AgCl reference electrode was used. Sample preparation was done by a dilution of apple juices with deionized water (1:1000 *v*/*v*) and subsequently filtration with a 0.2 µm porous PTFE-filter. Individual sugars were identified according to the retention time and quantified using a mix standard of the four sugars using the Chromeleon 6.8 software package (Thermo Scientific Dionex).

### 3.4. Statistical Analysis

For descriptive statistics and analysis of variance (ANOVA and Kruskal-Wallis test) the R statistical environment [45] was used. Parametric data was subjected to ANOVA and followed by Tukey test (*p* ≤ 0.05), whereas non-parametric variables such as titratable acid, pH and some carbohydrates were analyzed using Kruskal-Wallis test (*p* ≤ 0.05) followed by Mann-Whitney *U* test with Bonferroni correction. Principal component analysis (PCA) was performed with Unscrambler Version 10.3 [46] and partial least square regression (PLSR) models were carried out using the NIRCal^©^ 5.4.3010 software package (BUCHI^®^ AG, Flawil, Switzerland). Cross validation (in groups of 36 samples) was used to validate the models for carbohydrates and the accuracy of the model is defined by SECV, as follows:

(2) $$SECV=\;\sqrt{\frac{\sum_{i=1}^{n}{\left(y_{i}-Y_{i}\right)}^{2}}{n-p}}$$

with *y_i_*, the measured value of the *i*th observation; *Y_i_*, the predicted value of the left out spectra; *n*, the number of observations in the calibration set and *p*, the number of coefficients (number of secondary latent variables). For all the other parameters two third of the dataset were randomly used for calibration and one third for validation. Extreme outliers were removed from the data set. The accuracy of the calibration and validation models are defined by SEC and SEP, as follows:

(3) $$SEC=\;\sqrt{\frac{\sum_{i=1}^{n}{\left({ŷ}_{i}-y_{i}\right)}^{2}}{n-1}}$$

(4) $$SEP=\;\sqrt{\frac{\sum_{i=1}^{n}{\left({ŷ}_{i}-y_{i}-bias\right)}^{2}}{n-1}}$$

with *ŷ_i_*, the predicted value of the *i*th observation; *y_i_*, the measured value of the *i*th observation; *n*, the number of observations in the calibration set and the validation set, respectively and bias:

(5) $$bias=\frac{1}{n}\overset{n}{\underset{i=1}{\sum }}\left({ŷ}_{i}-y_{i}\right)$$

Wavelength selection was carried out automatically using an iterative calibration algorithm [47].

## 4. Conclusions

This study investigated the potential of NIRS as an analytical tool for the post-harvest management of fruit quality beyond present applications. Using apple as a model fruit, we composed a real-market sample set with two cultivars, different harvest and storage conditions. Our study complements previous work, but on a comprehensive dataset along the postharvest chain, highlighting the potential of NIRS to identify cultivars and freshly picked *vs.* stored fruit. Interestingly, we were able to identify for the first time the sun-exposed side of apples with its increased content of nutrients and functional metabolites, and to describe improved prediction models for established quality parameters. Analytical tools like NIRS, electronic noses and tongues have generated significant interest in food quality control for they provide fast, non-invasive, green (reagent-free) alternatives to traditional wet chemical methods. Our study shows that the potential of NIRS in post-harvest management is far from fully explored; however, the technology has its intrinsic limitations like the poor penetration depth. One promising approach is the combination of NIRS with other analytical techniques to satisfy the demand for efficient, reagent free analytical tools in the post-harvest management.

## Supplementary Materials

Supplementary materials can be accessed at: http://www.mdpi.com/1420-3049/20/08/13603/s1.
