# Supplementary material for: A Near Infrared Spectroscopy (NIRS) and Chemometric Approach to Improve Apple Fruit Quality Management: A Case Study on the Cultivars “Cripps Pink” and “Braeburn”
Source: Molecules. 2015 Aug 24;20(8):13603–19. doi: 10.3390/molecules200813603 (PMC6331841; doi:10.3390/molecules200813603)
Supplement: Supplementary file 1 [file molecules-20-13603-s001.pdf]

Supplementary Material

Table S1. Post-harvest evolution of quality parameters (mean) of "Braeburn" and "Cripps Pink" apples from the harvest date 2 (HT2) during long-term CA storage.

Variety	"Braeburn"						"Cripps Pink"					
**analysis time-point**	**0**	**7**	**15**	**21**	**28**	**32**	**0**	**6**	**15**	**20**	**27**	**30**
**sample number**	30	30	29	30	29	30	30	30	29	30	29	29
**weight [g] ***	209.5	205.8	211.8	195.4	201.2	204.2	211.1	214.0	207.8	214.3	213.0	203.9
**total juice [mL] ***		123.8 ^{a}	127.8 ^{a}	99.5 ^{b,c}	94.2 ^{c}	117.4 ^{a,b}		129.1 ^{a}	120.1 ^{a,b}	107.2 ^{b,c}	103.2 ^{c}	111.8 ^{b,c}
**pH $^{\#}$**	3.54 ^{a,b}	3.52 ^{a}	3.62 ^{c}	3.58 ^{b}	3.65 ^{c}		3.49 ^{a}	3.59 ^{b}	3.70 ^{c}	3.67 ^{c}	3.77 ^{d}	3.84 ^{e}
**TA [g/L malic acid] $^{\#}$**	5.3 ^{a}	5.2 ^{a,b}	4.8 $^{b.c}$	4.7 ^{b,c}	4.4 ^{c}		5.1 ^{a}	4.6 ^{b}	3.9 ^{c}	3.9 ^{c}	3.4 ^{d}	3.2 ^{d}
**TSS [°Brix] ***	9.9 ^{a}	12.4 ^{b}	12.1 ^{b}	12.1 ^{b}	12.7 ^{b}		13.3 ^{a}	11.9 ^{c}	12.8 ^{a,b}	12.2 ^{b,c}	13.2 ^{a}	12.9 ^{a,b}
**F_f [N] ***	86.7 ^{a}	82.6 ^{a,b}	76.8 ^{b,c}	71.5 ^{c,d}	69.3 ^{d}	72.1 $^{c.d}$	105.0 ^{a}	93.2 ^{b}	84.5 ^{c}	83.9 ^{c}	81.6 ^{c}	
**D [mm] ***	3.55 ^{a,b}	3.56 ^{a,b}	3.33 ^{a,b}	3.51 ^{a,b}	3.25 ^{a}	3.63 ^{b}	4.90 ^{a,b}	4.60 ^{b,c}	5.20 ^{a}	4.33 ^{c}	4.30 ^{c}	
**W_f [J] ***	0.18 ^{a}	0.16 ^{a,b}	0.15 ^{b,c}	0.15 ^{b,c}	0.13 ^{c}	0.16 ^{a,b}	0.29 ^{a}	0.25 ^{b}	0.22 ^{b,c}	0.21 ^{c}	0.20 ^{c}	
**F_{LC} [N] ***	63.2 ^{a}	60.4 ^{a,b}	58.3 ^{a,b}	54.9 ^{b,c}	51.0 ^{c}	55.2 ^{b,c}	92.2 ^{a}	77.0 ^{b}	66.3 ^{c}	63.2 ^{c}	57.5 ^{d}	
**S [N/mm] ***	39.6 ^{a,b}	47.5 ^{a}	32.3 ^{b}	37.0 ^{a,b}	47.7 ^{a}	35.8 ^{a,b}	37.0 ^{a}	32.2 ^{a,b}	55.6 ^{c}	28.1 ^{b}	31.0 $^{a.b}$	
**glucose [g/100 g] $^{\#}$**		0.8	1.0	1.1	1.0	1.2		0.5	0.6	0.6	0.6	0.6
**xylose [g/100 g] ***		0.03 ^{a}	0.04 ^{a,b}	0.05 ^{b}	0.06 ^{b}	0.05 ^{b}		0.04 ^{a}	0.05 ^{a,b}	0.06 ^{b}	0.06 ^{b}	0.06 ^{b}
**sucrose [g/100 g]$^{\#}$**		2.0	1.8	1.6	1.4	1.4		3.6 ^{a}	3.2 ^{a,b}	3.2 ^{a,b}	2.6 ^{b,c}	2.4 ^{c}
**fructose [g/100 g] ***		3.4	3.5	3.5	3.2	2.6		3.0	3.1	2.8	2.8	2.8

Means with different superscript letters in the same row differ significantly ($p < 0.05$) within one cultivar; * ANOVA followed by the Tukey test; $^{\#}$ Kruskal-Wallis test followed by the Mann-Whitney *U* test with Bonferroni correction.

Table S2. Post-harvest evolution of quality parameters (mean) of "Braeburn" and "Cripps Pink" apples from the harvest date 3 (HT3) during long-term CA storage.

**Variety**	**"Braeburn"**						**"Cripps Pink"**					
**analysis time-point**	**0**	**7**	**15**	**21**	**28**	**32**	**0**	**6**	**15**	**20**	**27**	**30**
**sample number**	30	28	28	29	27	29	30	30	30	30	30	29
**weight [g] ***	208.8	214.3	220.9	218.9	214.2	213.2	215.4	220.4	220.9	215.8	205.7	202.7
**total juice [mL] ***		127.1 ^{a}	130.7 ^{a}	115.6 ^{a,b}	101.4 ^{b}	121.5 ^{a,b}		128.1 ^{a}	130.6 ^{a}	115.7 ^{a,b}	104.3 ^{b}	111.7 ^{b}
**pH #**	3.58 ^{a}	3.62 ^{a,b}	3.65 ^{b,c}	3.62 ^{a,b}	3.69 ^{c}	3.81 ^{d}	3.49 ^{a}	3.56 ^{b}	3.67 ^{c}	3.66 ^{c}	3.82 ^{d}	3.85 ^{d}
**TA [g/L malic acid] #**	4.6 ^{a}	4.8 ^{a}	4.5 ^{a}	4.5 ^{a,b}	4.1 ^{b}	3.4 ^{c}	5.4 ^{a}	4.8 ^{b}	4.2 ^{c}	4.0 ^{c}	3.3 ^{d}	3.3 ^{d}
**TSS [°Brix] ***	12.2	13.2	12.3	12.7	13.0	12.9	13.2 ^{a}	12.6 ^{b}	13.1 ^{a,b}	12.9 ^{a,b}	13.0 ^{a,b}	12.6 ^{b}
**F_f [N] ***	87.2 ^{a}	79.6 ^{a,b}	79.0 ^{b}	72.6 ^{b,c}	67.5 ^{c}	65.0 ^{c}	94.4 ^{a}	92.9 ^{a}	85.6 ^{b}	79.7 ^{b,c}	74.1 ^{c}	
**D [mm] ***	3.68 ^{a,b}	3.75 ^{a}	3.39 ^{a,b}	3.40 ^{a,b}	3.27 ^{b}	3.54 ^{a,b}	5.01 ^{a}	4.99 ^{a}	6.22 ^{b}	4.74 ^{a}	4.51 ^{a}	
**W_f [J] ***	0.18 ^{a}	0.17 ^{a,b}	0.16 ^{a,b,c}	0.15 ^{b,c}	0.13 ^{c}	0.14 ^{b,c}	0.28 ^{a}	0.28 ^{a}	0.28 ^{a}	0.23 ^{b}	0.20 ^{b}	
**F_{LC} [N] ***	63.8 ^{a}	60.8 ^{a}	58.9 ^{a,b}	53.5 ^{b,c}	52.3 ^{b,c}	52.0 ^{c}	84.2 ^{a}	82.7 ^{a,b}	76.1 ^{b,c}	70.4 ^{c}	62.2 ^{d}	
**S [N/mm] ***	33.5 ^{a}	44.6 ^{b}	32.3 ^{a}	35.0 ^{a}	54.4 ^{b}	34.8 ^{a}	36.9	32.1	30.9	31.2	28.3	
**glucose [g/100 g] #**		0.8 ^{a}	1.0 ^{a,b}	1.0 ^{a,b}	1.0 ^{a,b}	1.3 ^{b}		0.4 ^{a}	0.5 ^{a}	1.1 ^{b}	0.5 ^{a}	0.5 ^{a}
**xylose [g/100 g] ***		0.02 ^{a}	0.03 ^{a,b}	0.05 ^{c}	0.05 ^{b,c}	0.03 ^{a,b}		0.02 ^{a}	0.04 ^{a,b}	0.06 ^{b,c}	0.05 ^{b,c}	0.06 ^{c}
**sucrose [g/100 g] #**		2.1 ^{a}	1.6 ^{a,b}	1.9 ^{a,b}	1.2 ^{b}	3.3 ^{c}		3.5 ^{a}	3.8 ^{a}	2.4 ^{b}	2.1 ^{b}	2.3 ^{b}
**fructose [g/100 g] ***		3.0 ^{a}	3.0 ^{a}	3.0 ^{a}	2.9 ^{a}	1.3 ^{b}		2.7 ^{a,b,c}	3.1 ^{b,c}	3.3 ^{c}	2.2 ^{a}	2.5 ^{a,b}

Means with different superscript letters in the same row differ significantly ($p < 0.05$) within one cultivar; * ANOVA followed by the Tukey test; # Kruskal-Wallis test followed by the Mann-Whitney *U* test with Bonferroni correction.

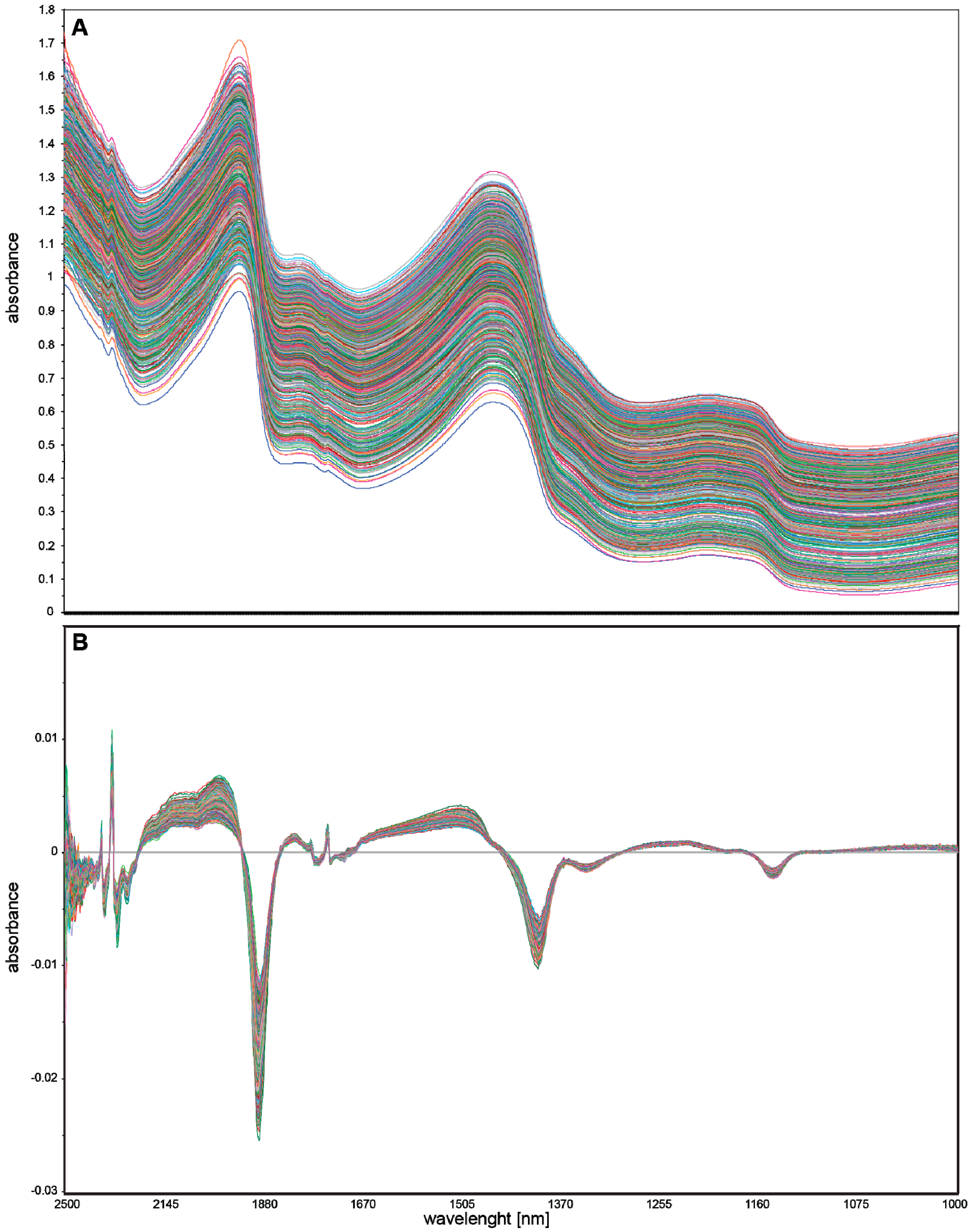

Figure S1. Raw NIR spectra (**A**) and spectra pretreated by de-trending and first derivative Savitzky-Golay (**B**) for 1049 apples.

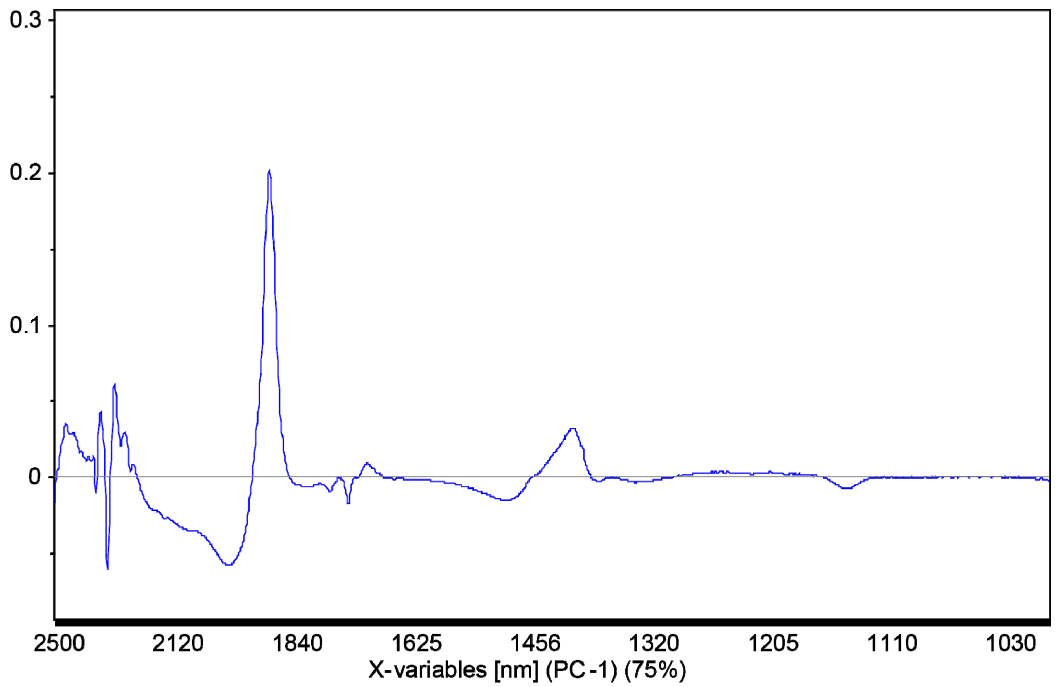

Figure S2. PCA loadings plot of NIR data acquired from the complete data set (515 "Braeburn" and 534 "Cripps Pink" apples).
